# Electrochemical Oxidation as a Tool for Generating Vitamin D Metabolites

**DOI:** 10.3390/molecules24132369

**Published:** 2019-06-26

**Authors:** Laura Navarro Suarez, Sonja Thein, Constanze Kallinich, Sascha Rohn

**Affiliations:** University of Hamburg, Hamburg School of Food Science, Institute of Food Chemistry, Martin-Luther-King-Platz 6, 20146 Hamburg, Germany

**Keywords:** electrochemical oxidation, EC-MS, ergocalciferol, cholecalciferol, metabolism

## Abstract

The electrochemical behavior of the vitamers cholecalciferol and ergocalciferol was investigated in order to determine whether it is possible to evaluate phase-I and phase-II metabolism of these steroids and yield metabolites that can serve as reference material. The vitamers were electrochemically-oxidized using an electrochemical system (ROXY™ EC system). The influence of pH value, solvent, and potential was evaluated. When using methanol or ethanol, the formation of artificial methoxy or ethoxy groups, respectively, was observed, while the use of acetonitrile did not show any formation of further functional groups. A neutral pH value and use of a constant potential led to the highest number of oxidation products with intensive signals. Additionally, a binding study between vitamin D and glucuronic acid as an example for phase-II conjugation was carried out. It was possible to detect adduct formation. Coupling mass spectrometry directly to electrochemistry (EC-MS) is a promising approach for generating vitamin D metabolites and/or yielding a number of metabolites without in vivo or in vitro test systems. It can support or even replace animal studies in the long-term and might be promising for yielding reference compounds.

## 1. Introduction

Vitamin D or calciferols are substances from the group of secosteroids. The two bioequivalent forms of calciferol represent vitamin D_2_ (ergocalciferol) and vitamin D_3_ (cholecalciferol) [1]. Their main functions are the regulation of calcium homeostasis or gene expression in the parathyroid gland [2]. In addition, they trigger the expression of genes that regulate the immune system and are responsible for the differentiation of epidermal and hematopoietic cells. A vitamin D deficiency can lead to mineralization disorders of the skeleton (rickets) [3]. Cholecalciferol is mainly obtained by exposure of the skin to UV-B radiation from sunlight. The B-ring of the sterane backbone of provitamin D_3_ is photolytically cleaved at position C9/C10 to form previtamin D_3_. Previtamin D_3_ is thermodynamically unstable and isomerizes to the more stable form named vitamin D_3_. The reaction of 7-dehydrocholesterol to previtamin D_3_ is induced in the wavelength range of 280–320 nm [4]. Cutaneous synthesis can be influenced by various molecules that also absorb radiation (e.g., melanins, proteins, sunscreens, or clothing) [4,5,6]. In an analogous way, ergocalciferol is formed by the interaction of UV-B radiation with ergosterol. Due to the ability of endogenous biosynthesis, the D-vitamin terminology seems to be old-fashioned, and assignment as (pro)-hormones would be more appropriate [7]. Ergocalciferol and cholecalciferol are not the main biological active metabolites in the organism. Consequently, a transformation occurs. This takes place at different regulation levels. Cholecalciferol is transported via the blood into the liver linked to vitamin D binding protein (DBP). There it gets converted to 25-hydroxycholecalciferol (calcidiol), the major circulating form of vitamin D, which is used to define nutritional vitamin D status. Bound to DBP, calcidiol is transported via the blood to the kidneys where a further hydroxylation occurs. The final activating ligand for the vitamin D receptor is 1α,25-dihydroxycholecalciferol (calcitriol) [8]. The metabolism from vitamin D to calcitriol is regulated by enzymes of the cytochrome-P450-superfamily. This especially concerns the enzyme 1α-hydroxylase (1α-OHase) [5]. While cyclic adenosine monophosphate (cAMP) induces the transcription of the enzyme, phosphate and calcium ions as well as calcitriol inhibit them. In addition, regulation via parathyroid hormone (PTH), a hormone that is responsible for calcium homeostasis, is also involved, illustrating the complexity of the whole regulation process. At a low serum calcium level, PTH is secreted by the parathyroid gland. It is taken up by PTH receptors and activates the adenylate cyclase and thus the transcription of 1α-OHase. Furthermore, a calcium sensor protein affects the activity of 1α-OHase [9].

Vitamin D_2_ is mainly taken up with food and present in mushrooms and yeasts. Vitamin D_3_-rich foods are, for example, egg yolk or fish with high lipid contents (herring, mackerel) [10]. However, people with an increased risk of vitamin D deficiency are those with inadequate sun exposure, impaired vitamin D intake or absorption [11]. According to the German Society for Nutrition (DGE), intake of vitamin D supplements is not sufficient to reach the estimate of adequate intake in the absence of endogenous synthesis, which will provide the desired supply of at least 50 nmol/L 25-hydroxyvitamin D (serum concentration). The difference must be therefore covered by enhanced endogenous synthesis or by the intake of vitamin D supplements [12]. To gain the advantages of a drug or dietary supplement, absorption, distribution, metabolism, and elimination of the active substance has to be taken into account. Metabolism is an essential component in pharmacokinetics because the excess of a drug needs to be excreted. The conversion of a bioactive compound to a metabolite of different bioactivity significantly influences its effects and sustainability. Phase-I and phase-II metabolism are crucial steps in this process. While phase-I metabolism includes oxidative, reductive, and hydrolytic reactions, phase-II metabolism involves the formation of bonds between the drug and small hydrophilic molecules (conjugation with e.g., glutathione, sulfate, glucuronic acid). The final step of this entire process is elimination/excretion. The hydrophilic molecule is excreted from the organism via different ways [13].

Due to a high prevalence of vitamin D deficiency in the Northern European population, an intake of vitamin D supplements is conceivable. To ensure goal-oriented and cost-effective research and development, it is therefore of certain importance to elucidate the pharmacokinetics and metabolism of potentially new vitamin D preparations or vitamin D-enriched food matrices at an early stage [14,15]. Conventional metabolism studies are based either on in vitro cell culture experiments, usually using hepatic or kidney cells, or cell extracts [16]. More comprehensively, metabolism is tested with the help of in vivo tests, following interventions (in animal or even human experiments) with the application of foods or drug preparations. With regard to Article 4 of the European Commission Directive 2010/63/EU on the protection of animals used for scientific purposes, it is intended to avoid, reduce, or improve procedures for animals used in animal studies [17]. Promisingly, the application of electrochemistry directly coupled to mass spectrometry (EC-MS) could be a tool for simulating the endogenous metabolism of vitamin D or yielding metabolites (for follow-up analyses).

EC-MS represents a relatively new approach that allows investigations of endogenous oxidative processes in the organism by simulation. In the past there were many studies that used EC-MS for metabolomics, proteomics, and genomics approaches, as well as in fields of environmental analysis, forensics, and toxicology [18,19,20]. It can be assumed that the approach is capable of mimicking selective redox processes of the cytochrome-P450 enzyme family (CYP). For example, aliphatic, allylic, aromatic, and benzylic hydroxylations, *N-*, *O-*, and *S*-dealkylations, *N-* and *S*-oxide formation, as well as dehydrogenations have been successfully simulated [19]. Established methods for in vitro simulations are based on the use of CYP-enzymes of the liver and thus, on animal tissues or animal experiments. In these approaches, reproducibility is limited, because metabolism is dependent on the expression levels and activity of CYP450 enzymes. However, while the most important metabolites have been known for decades, enzyme promiscuity might also lead to further, less studied metabolites [21]. Other disadvantages of the traditional methods represent the enormous expenditure of time and associated high costs, which usually occur when carrying out intervention studies [19]. Isolating compounds in appropriate yields from in vivo sample material is laborious and might raise ethical issues.

Therefore, the aim of this study was to investigate the electrochemical behavior of the two D vitamers ergocalciferol and cholecalciferol and to investigate whether it is possible to evaluate phase-I and phase-II metabolism of these essential molecules or if the results are different to the metabolites described in the literature. There are also still a number of metabolites that have not yet been fully elucidated in vivo, as matrix and concentration might have been too low for a comprehensive study. A different approach for obtaining metabolites is evaluated.

## 2. Results and Discussion

### 2.1. Electrochemical Oxidation of Vitamin D by Means of EC-ESI-MS

At first, the electrochemical parameters for the highest conversion rate of ergocalciferol and cholecalciferol were assessed. For this purpose, a solution containing 100 µM ergocalciferol or 100 µM cholecalciferol was electrochemically oxidized. The pH values of the solutions were 2.0, 6.0, 7.4, and 8.0. The potential was raised from 0.0 V to 3.0 V in steps of 0.3 V (for ergocalciferol) or 0.5 V (for cholecalciferol) using a ROXY™ potentiostat with a thin-layer µ-PrepCell, consisting of a boron-doped diamond electrode, a titanium counter electrode, and a palladium/hydrogen reference electrode (Antec Leyden B.V., Leiden, The Netherlands). The 25-hydroxylation takes place in the liver; consequently, an optimal electrochemical in vitro simulation is expected to be at a pH value of 8.0 [22]. It was found that a comparable oxidation behavior could be achieved at the different pH values of 6.0, 7.4, and 8.0, while high acidity (pH 2.0) led to non-reproducible mass spectra.

**Figure 1:**
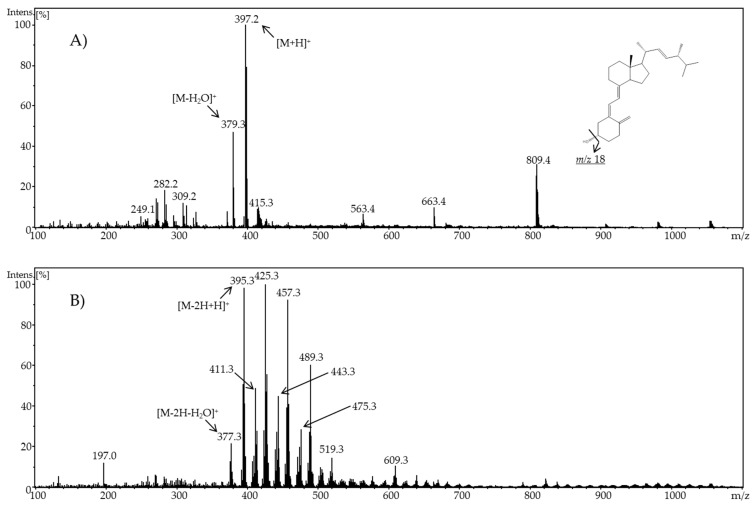
Full scan mass spectra of (**A**) pure ergocalciferol and (**B**) electrochemically-oxidized ergocalciferol using a potential of ϕ = 1.8 V at pH 7.4.

For ergocalciferol, preliminary signals of potential oxidation products could be observed at a potential of 0.9 V. A potential of 1.8 V and a neutral pH value led to the highest number of oxidation products with intensive signals. In Figure 1, a comparison of the resulting mass spectra of ergocalciferol at 0.0 V and 1.8 V at pH 7.4 is shown. In the resulting ESI mass spectrum in positive ion mode of pure ergocalciferol (molar mass 396.6 g/mol), various signals can be observed. Ergocalciferol formed quasi-molecular ions [M + H]^+^ (*m/z* 397.2) as well as ions resulting from a neutral loss of water (*m/z* 379.3). The last reaction is also a typical reaction for bile acids in methanol in positive ESI ion mode [23]. When adjusting a potential of 1.8 V, additional signals could be observed in the resulting mass spectrum. Mass differences greater than 2 Da suggested a successful oxidation. A decrease of the molecular mass by 2 Da indicated an oxidation of a hydroxyl group to form a keto group, for example. There were, amongst other signals, new signals with *m/z* 377.3, *m/z* 393.3, *m/z* 395.3, *m/z* 425.3, and *m/z* 457.3. Single or double hydroxylation of ergocalciferol could explain the signals at *m/z* 395.3, *m/z* 377.3, and *m/z* 393.3. The decrease of the molecular peak from 2 Da to *m/z* 395.3 could also be due to the oxidation of a hydroxyl group to a keto group or from dehydration of an alkane to an alkene. The resulting ESI mass spectrum of electrochemically-oxidized ergocalciferol could thus show signals of the typical metabolites 25-hydroxyergocalciferol, 1,25-dihydroxyergocalciferol, 24-hydroxyergocalciferol, 1,24-dihydroxyergocalciferol, and 1,24,25-trihydroxyergocalciferol. As these products could not be identified only by their *m/z* signals, the mass spectrum alone is not meaningful for determining which compound is specifically involved.

**Figure 2:**
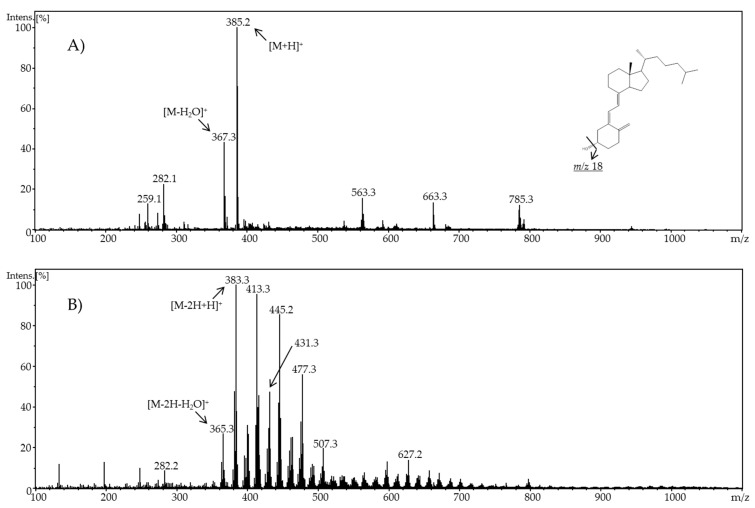
Full scan mass spectra of (**A**) pure cholecalciferol and (**B**) electrochemically-oxidized cholecalciferol using a potential of ϕ = 2.0 V at pH 7.4.

For cholecalciferol, preliminary signals of potential oxidation products could be observed when applying a potential of 1.5 V. A potential of 2.0 V and a neutral pH value led to the highest number of oxidation products with intensive signals. In Figure 2, a comparison of the resulting mass spectra of cholecalciferol at 0.0 V and 2.0 V at pH 7.4 is shown.

In the resulting ESI mass spectrum in positive ion mode of cholecalciferol (molar mass 384.6 g/mol), diverse signals can be found. The oxidation behavior of cholecalciferol was similar to that of ergocalciferol. Cholecalciferol also formed quasi-molecular ions [M + H]^+^ (*m/z* 385.2) as well as ions resulting from neutral water loss (*m/z* 367.3). When adjusting a potential, many new signals were detected. Mass differences of 2 Da suggested a successful oxidation. A decrease of the molecular mass by 2 Da indicated an oxidation of a hydroxyl group forming a keto group or dehydration from an alkane to an alkene, for example. The increase of 14 Da, 16 Da, or 18 Da compared to the initial cholecalciferol indicated additional oxygen molecules, e.g., in the form of keto or hydroxyl groups. For an in-depth elucidation of the products generated during the electrochemical oxidation of cholecalciferol, further methods have to be applied e.g., tandem-MS or NMR.

### 2.2. Influence of the Composition of the Solvent

When using methanol as part of the solvent for the electrochemical oxidation of vitamin D, the formation of methoxy groups were observed. This could be explained with a mass difference of 32 Da in MS^2^. To verify this assumption, use of ethanol and acetonitrile in the solvent was examined. Mass spectrometric detection should reveal how the oxidation behavior of vitamin D_2_ and vitamin D_3_ changed. The replacement of methanol with ethanol in the mass spectrometer was expected to cause the neutral fragmentation of ethanol instead of methanol. The fragmentation of ethanol would be due to an ethoxy group being formed in the electrochemical oxidation. When using the solvent acetonitrile, neither methoxy nor ethoxy groups were formed.

**Figure 3:**
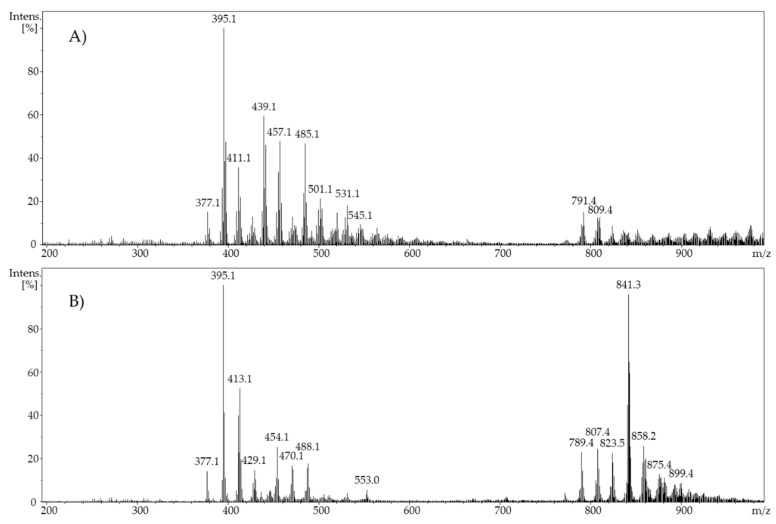
Full scan mass spectra of (**A**) ergocalciferol in ethanol/water (80/20, *v*/*v*), pH 7.4, potential 2 V and (**B**) ergocalciferol in acetonitrile/water (50/50, *v*/*v*), potential 1.2 V.

Based on the resulting ESI mass spectrum and various tandem mass spectra of selected fragments of an electrochemically-oxidized ergocalciferol in a solution of ethanol/water at a potential of 2.0 V, it could be observed that many oxidation products had mass losses of 46 Da. Those mass losses of 46 Da showed that the electrochemical oxidation of vitamin D_2_ produced ethoxy groups. These ethoxy groups result in fragmentation patterns in the form of ethanol (molar mass: 46 g/mol). While using acetonitrile as a part of the solvent, neither mass losses of 32 Da (methanol) nor 46 Da (ethanol) could be observed. Instead, a mass increase of 16 Da, which indicates the formation of hydroxyl groups, was determined. A comparison of the resulting mass spectra is shown in Figure 3.

### 2.3. Identification of Oxidized Metabolites via LC-ESI-MS

**Figure 4:**
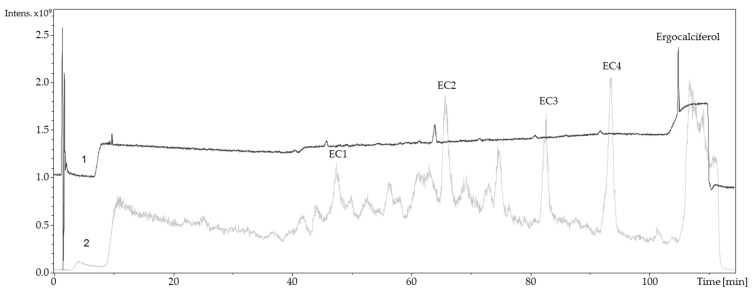
Liquid chromatography coupled to electrospray ionization mass spectrometry and diode-array detection (LC-ESI-MS/DAD) chromatogram of electrochemical oxidized ergocalciferol (2 mM). ECx: Shortcut for ergocalciferol oxidation products. 1: Ergocalciferol, oxidized, UV chromatogram 190–800 nm; 2: Ergocalciferol, oxidized, total ion chromatogram (TIC + All MS).

To identify and/or characterize the oxidized products resulting from the electrochemically-induced oxidation, a LC-ESI-MS/DAD method was used for evaluation. For that approach, vitamin D was successfully oxidized in a synthesis cell to generate higher yields of oxidation products. Afterwards, the resulting solution was injected into an UHPLC system. Detection was carried out by an ESI mass spectrometer in positive ion mode and a DAD. LC-MS chromatograms are shown in Figure 4 and Figure 6. The chromatograms show that numerous oxidation products are formed during electrochemical oxidation. Because of their high signal intensity, four products of each metabolite were studied in detail (abbreviated EC1–EC4, CC1–CC4). The MS spectra of EC1–EC4 and CC1–CC4 were recorded (Table 1).

**Figure 5:**
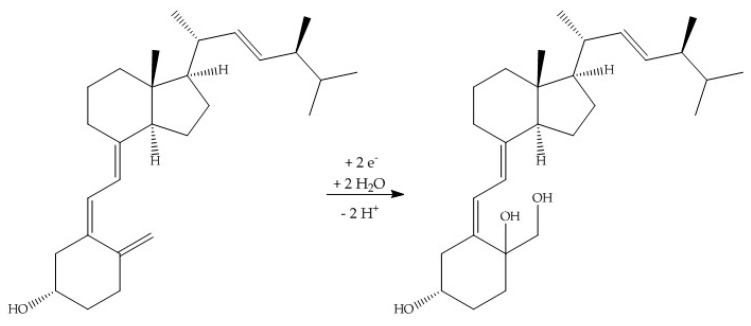
Proposed hydroxylation of ergocalciferol, leading to a 10,19-hydroxylated ergocalciferol (molar mass: 430.7 g/mol).

The relatively high UV-signal of ergocalciferol in comparison to the oxidation products was remarkable. Consequently, it was assumed that a reaction took place at the conjugated double bond system of ergocalciferol during the oxidation. The fragmentation pattern of EC1 (quasimolecular ion *m/z* 431.1) showed mass losses of 18 Da, 36 Da, and 54 Da which indicated a single, a double, and a triple dehydration. An increase of 34 Da could be explained with a two-fold hydroxylation at an unsaturated carbon. The ^1^H-NMR spectrum of EC1 revealed a loss of signals compared to the ^1^H-NMR spectrum of ergocalciferol. While ergocalciferol showed two signals with a chemical shift close to 5 ppm, those signals were absent in EC1. Consequently, it can be assumed that a two-fold hydroxylation took place at the conjugated triene-system at C19 (Figure 5). In the literature, such a metabolite is not described yet. The mass spectrum of EC2 showed signals at *m/z* 411.1 (quasimolecular ion), *m/z* 821.4 (dimer [2M + H]^+^), *m/z* 425.1, and *m/z* 457.1. EC3 and EC4 showed similar mass spectra and fragmentation patterns. Both had quasimolecular ions with *m/z* 427.1 and mass losses that indicated water elimination and formation of methoxy groups.

**Figure 6:**
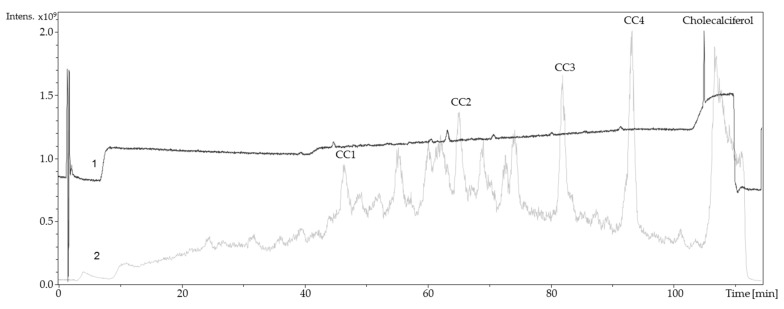
LC-MS/DAD chromatogram of electrochemical oxidized cholecalciferol (2 mM). CCx: Shortcut for cholecalciferol oxidation products; 1: Cholecalciferol, oxidized, UV chromatogram 190–800 nm; 2: Cholecalciferol, oxidized, TIC + All MS.

The total ion chromatogram and the UV-chromatogram of cholecalciferol (Figure 6) were similar to those of ergocalciferol. They showed many signals that represent the generated oxidation products. The quasimolecular ions of the four oxidation products of cholecalciferol (CC1–CC4) were each 12 Da higher than EC1–EC4. This can be explained by the 12 g/mol difference of the molar masses of ergocalciferol and cholecalciferol.

**Figure 7:**
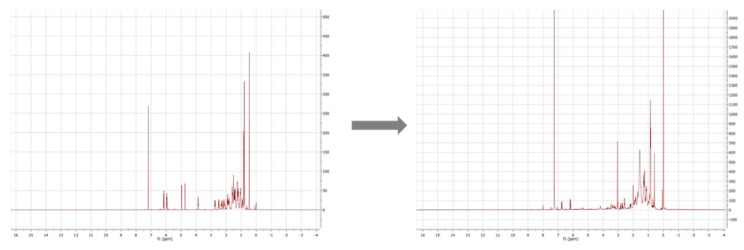
Comparison of ^1^H-NMR spectra of a commercially-available cholecalciferol reference (left) and the product CC1 (right).

However, this technique is limited by the semi-preparative HPLC system that was used, which was not able to separate and enrich the amount of the numerous oxidation products that could be investigated further. This can be explained with the structural similarities and the little differences of the products (e.g., *trans* or *cis* double bonds). Only a ^1^H-NMR spectrum of an oxidation product in a methanol solution indicated that the electrochemical oxidation involved a reaction at the double bond of the conjugated triene system. The singlet signal of the two protons at position C19, which were to be expected for chemical shifts between 4 ppm and 6 ppm, were no longer present in the spectrum (Figure 7).

Nevertheless, it can be assumed that an electrochemical oxidation took place. It was possible to simulate dehydrations, hydroxylations, and methoxylations or ethoxylations, respectively. The results from Nallbani et al., which also deal with a characterization of those oxidation products, showed that the electrochemical oxidation presumably takes place at the delocalized electron system at C8–C19. A reaction with the less reactive aliphatic chain (C20–C27) would be unlikely [24]. Filik et al. suspected the generation of 25-hydroxycalciferol [25]. On the other hand, the results from the present study showed the generation of another metabolite. However, the chemical structure has to be verified. To identify and characterize the generated oxidation products, further NMR experiments is recommended, needing far higher amounts of the single compounds.

### 2.4. Investigation of Adduct Formation of Vitamin D and Glucuronic Acid Using EC-ESI-MS

For evaluating the feasibility of simulating the phase-II metabolism of vitamin D derivatives and investigating the binding properties and the reactivity of the oxidation products, a reaction of the electrochemically-oxidized ergocalciferol and cholecalciferol with glucuronic acid was initiated via a reaction coil. Glucuronic acid (GlA) is an antioxidant and detoxifying agent which binds to 25-hydroxyvitamin D_3_ to form 25-OH-D_3_-25-glucuronide, 25-OH-D_3_-3-glucuronide, and 5,6-*trans*-25-OH-D_3_-25-glucuronide [26] and binds to 25-hydroxyvitamin D_2_ to form 25-OH-D_2_-25-glucuronide and 25-OH-D_2_-3-glucuronide. These formations increase the hydrophilic properties for improved excretion. Defined volumes of both reactants (100 µM calciferol and 1 mM GlA) were injected into the electrochemical system via a syringe pump. After passing through a reaction coil, the reaction products were directly detected using an ESI-MS ion trap mass analyzer in positive ion mode for identifying potential adducts.

**Figure 8:**
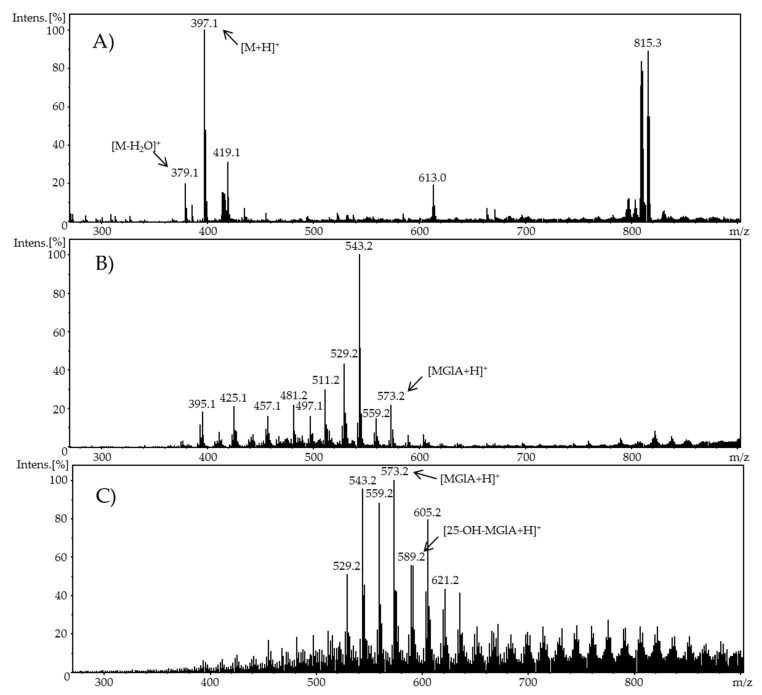
Full scan mass spectra of (**A**) the potential adduct formation of ergocalciferol (100 µM) and glucuronic acid (1 mM) at 0.0 V (pH 7.4), (**B**) the potential adduct formation of ergocalciferol and glucuronic acid at 1.8 V, and (**C**) the potential adduct formation of ergocalciferol and glucuronic acid at 3.0 V.

The resulting full scan mass spectrum of the pure ergocalciferol and GlA mixture (Figure 8A), the full scan mass spectrum of oxidized ergocalciferol mixed with glucuronic acid at an adjusted potential of 1.8 V (Figure 8B), and the full scan mass spectrum of oxidized ergocalciferol mixed with GlA at 3.0 V (Figure 8C) showed diverse new signals indicating adduct formations. In the mass spectrum of ergocalciferol and GlA, signals of free ergocalciferol ([M + H]^+^, *m/z* 397.1; [M − H_2_O]^+^, *m/z* 379.1) were observed.

There were no signals of free glucuronic acid, because its molar mass (194.1 g/mol) or its *m/z*, respectively, is below the detected range. When adjusting a potential of 1.8 V, the resulting mass spectrum showed different signals. The signals of free ergocalciferol disappeared and new signals emerged. The signal with *m/z* 573.2 is suggested to derive from ergocalciferolglucuronide ([MGlA + H]^+^). The signal with *m/z* 543.2 is assumed to represent a fragment of ergocalciferolglucuronide. The resulting mass spectrum of GlA and electrochemically-oxidized ergocalciferol at a potential of 1.8 V primarily showed the formation of ergocalciferolglucuronide. With regard to this observation, the binding behavior at a potential of 3.0 V was also investigated. In addition to ergocalciferolglucuronide, further conjugates with GlA and various oxidation products were formed. The GlA-conjugate with an *m/z* of 589.2 could be detected with a higher signal intensity. In addition, a GlA-conjugate was detected with *m/z* 605.2. Signals with *m/z* 589.2 and *m/z* 605.2 could result from the conjugation of an ergocalciferol oxidation product (e.g., 25-hydroxyergocalciferol) and GlA. The signal with *m/z* 589.2 could derive from the conjugate 25-OH-D_2_-glucuronide, which has already been identified in vivo [26,27]. Only a few signals with a mass difference of 2 Da relating to pure ergocalciferol were observed at a potential of 3.0 V. As described above, those signals can be assigned to oxidized ergocalciferol (e.g., *m/z* 395.1).

Due to the fact that the ESI mass spectra represented a mixture of the formed oxidation products and their conjugation with GlA, only tentative structures can be given. Using LC-MS/MS, the generated GlA-conjugates could be more accurately characterized. The separation of the products on a preparative scale for further NMR experiments could contribute to the absolute structure elucidation of the products. Nevertheless, it can be assumed that the detected *m/z* represent the known metabolites from phase-II metabolism of vitamin D.

## 3. Materials and Methods

### 3.1. Chemicals and Materials

Cholecalciferol, ergocalciferol, 25-hydroxyergocalciferol, 1α,25-dihydroxyergocalciferol, and ammonium formate were purchased from Sigma-Aldrich GmbH (Steinheim, Germany). The compound 24,25-dihydroxyergocalciferol was purchased from IsoSciences LLC (Ambler, PA, USA). Methanol and acetonitrile were purchased from Carl Roth GmbH & Co. KG (Karlsruhe, Germany). Formic acid was purchased from VWR International GmbH (Darmstadt, Germany) and D(+)-glucuronic acid sodium salt monohydrate was purchased from Merck Chemicals GmbH (Darmstadt, Germany). All of the chemicals were used at the highest quality available. Water was purified before utilization via the Direct-Q 3 UV-R system (Merck Chemicals GmbH, Darmstadt, Germany).

### 3.2. Electrochemical Oxidation of Vitamin D by Means of Electrochemistry Directly Coupled to Electrospray Ionization Mass Spectrometry (EC-ESI-MS)

**Figure 9:**
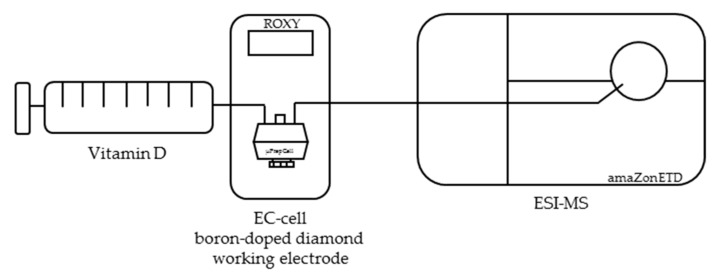
Scheme of the instrumental setup for the electrochemical oxidation of vitamin D using an electrochemistry directly coupled to electrospray ionization mass spectrometry (EC-ESI-MS) system. Vitamin D is oxidized electrochemically via a thin layer cell (µ-PrepCell) consisting of a boron-doped diamond working electrode and directly detected via electrospray ionization mass spectrometry (ESI-MS).

The electrochemical oxidation of vitamin D was performed using an electrochemical system (ROXY™, Antec Leyden B.V., Leiden, The Netherlands), which was equipped with a preparative thin-layer cell (µ-PrepCell, Antec Leyden B.V., Leiden, The Netherlands). The thin-layer cell consisted of a boron-doped diamond working electrode, a titanium counter electrode, and a palladium/hydrogen reference electrode. The electrochemical potential was controlled using a ROXY™ potentiostat (Antec Leyden B.V., Leiden, The Netherlands). A schematic of the instrumental setup is shown in Figure 9.

A solution containing 100 µM of cholecalciferol or ergocalciferol in methanol/water (90/10, v/v) and 20 mM formic acid was injected into the electrochemical system using an external syringe pump with a set flow rate of 10 µL/min. Moreover, the solvents ethanol (ethanol/water 80/20, v/v) and acetonitrile (acetonitrile/water 50/50, v/v) were tested. Four different solutions of varying pH values were prepared. The solutions were set to pH 2.0, pH 6.0, pH 7.4, and pH 8.0 (with diluted hydrochloric acid and ammonium hydroxide, respectively) to simulate the compartments of the stomach, intestine, blood, and bile. The total volume of µ-PrepCell (depending on an effective spacer thickness of 150 µm) was 11 µL. The temperature of the electrochemical cell during analysis was 23 °C. First, scan mode was applied to determine the electrochemical potential where the conversion rate was the highest (0.0 V to 3.0 V in 0.3 V steps for ergocalciferol and in 0.5 V steps for cholecalciferol). Afterwards, a constant potential was applied. The detection of oxidation products was performed with an ESI-MS ion trap mass analyzer in positive ion mode (amaZon speed ETD, Bruker Daltonik GmbH, Bremen, Germany), with the following mass spectrometer settings: ion spray voltage: 4.5 kV; ion source heater: 350 °C; source gas: 55 psi.

### 3.3. Identification of Oxidized Metabolites via LC-ESI-MS/DAD

For the determination of the type of oxidized vitamin D derivatives that were generated in the synthesis cell, the solution containing the potentially oxidized species was measured directly with LC-ESI-MS. Chromatographic separation was performed using a Dionex UltiMate™ 3000 ultra-high performance liquid chromatography (UHPLC) system (Thermo Fisher Scientific Inc., Waltham, MA, USA) equipped with a reversed-phase HPLC column (Phenomenex Kinetex^®^ 2.6 µm RP 18 100 Å, 150 × 2.1 mm) and a Kinetex^®^ C18 security guard column (Phenomenex Ltd. Deutschland, Aschaffenburg, Germany) using a constant flow of 200 µL/min. Mass spectrometric detection was performed by an ESI-MS ion trap mass analyzer (amaZon speed ETD, Bruker Daltonik GmbH, Bremen, Germany), recording mass spectra in positive ion mode. The injection volume was 5 µL, the column oven temperature was set to 30 °C, and the autosampler was kept at 10 °C. The reversed-phase chromatographic method consisted of a mobile phase system adapted from an existing method with some modifications for the optimized separation of oxidized bile acids [28]. The mobile phase A was water and B was acetonitrile/methanol (3/1, *v*/*v*), both containing 0.1% formic acid. The gradient elution started with 70% A for 4 min and then linearly decreased to 35% A within 1 min, which was kept constant for 33 min. Afterwards, composition was decreased to 30% A in 1 min. After a linear decrease to 15% A within 51 min, the percentage of A remained constant at 15% for 10 min. In 1 min, the percentage of A decreased to 0% and held for 5 min. Composition was brought back to an initial ratio of 70% A within 1 min, followed by 7 min of re-equilibration. The LC-MS system was controlled by Bruker Compass HyStar 3.2 (Bruker Daltonik GmbH, Bremen, Germany). Potentially generated oxidation products were identified via retention time, mass spectra and UV/VIS-spectra in comparison to commercially available vitamin D derivatives. For this purpose, solutions of 25-hydroxyergocalciferol, 1α,25-dihydroxyergocalciferol, and 24,25-dihydroxyergocalciferol (each at 0.04 mg/mL in methanol) were injected and analyzed with mass spectrometric and photometric detection.

### 3.4. Investigation of Adduct Formation of Vitamin D and Glucuronic Acid Using EC-ESI-MS

Phase-II metabolism was simulated by the adduct formation of ergocalciferol or cholecalciferol, respectively, and glucuronic acid as an example of phase-II conjugation. For this purpose, a second flow system containing a glucuronic acid solution with a concentration of 1 mM in methanol was added to the oxidized vitamin D solution. The ergocalciferol solution contained 100 µM ergocalciferol in methanol/water (90/10, v/v) and had a pH value of 7.4. Adjusted potentials were either 1.8 V and 3.0 V (ergocalciferol) or 2.0 V and 3.0 V (cholecalciferol). The flow rate of both syringe pumps (glucuronic acid and vitamer) was set to 20 µL/min. The temperature of the electrochemical cell during oxidation was 23 °C. The ratio of both analytes in the reaction coil was 1:1. After a reaction period in a 100 µL reaction coil and an incubation duration of 5 min, the mixture was infused directly into an ESI-MS ion trap mass analyzer (amaZon speed ETD, Bruker Daltonik GmbH, Bremen, Germany) in positive ion mode with the following settings: ion spray voltage: 4.5 kV; ion source heater: 350 °C; source gas: 55 psi.

## 4. Conclusions

In the present work, the electrochemical behavior of cholecalciferol and ergocalciferol was investigated. Thereby, it was found that both metabolites exhibit a similar electrochemical behavior. For ergocalciferol, a potential of 1.8 V and for cholecalciferol a potential of 2.0 V led to the highest number of oxidation products (metabolites) and an almost quantitative conversion.

When using methanol or ethanol as part of the solvent, methoxy or ethoxy groups, respectively, were generated. The characterization of the oxidation products should exhibit to which extent the metabolites of the secosteroids can be yielded electrochemically under these conditions. A comparison of retention times, UV signals, and mass spectra indicated that ergocalciferol and cholecalciferol had nearly identical oxidation behaviors. It can be concluded that no reaction took place at the double bond at C22–C23 of ergocalciferol. However, was be determined that the characteristic metabolites such as 25-hydroxyergocalciferol or 1α,25-dihydroxyergocalciferol were not the main products during the electrochemical oxidation. A complete metabolite profile was found. Consequently, it was possible to simulate dehydrations, hydroxylations, and methoxylations or ethoxylations, respectively. Therefore, this approach could be used for the generation of further metabolites that might be of lower concentration in vivo or in vitro. To simulate phase-II metabolism, a binding study with glucuronic acid was performed. Both the educts and the generated oxidation products bind to glucuronic acid. It was possible to generate products that are known from in vivo studies. A detailed clarification of the resulting chemical structures of the oxidation products and adducts was not possible at this time. However, the direct coupling to mass spectrometry and the use of NMR spectroscopy allowed the suggestion of tentative structures. In the future, an enrichment of the oxidation products is recommended so that there is enough yield for further experiments like 2D-NMR.

EC-MS offers numerous advantages. First and foremost, it should be mentioned that it can be a substitute for animal-based studies. Furthermore, in direct comparison to traditional methods, EC-MS requires less time and lower costs and underlies a more precise control of the experimental conditions. It seems to be possible to simulate different oxidation conditions, leading to further metabolites that can be compared to the in vivo formation mechanisms (e.g., with regard to their electrochemical redox potential value).

With regard to food or nutrition applications, this study might provide information for e.g., vitamin D-fortified food products. As vitamin D is added to corresponding food items as a preparation or pure compound(s), the newly identified oxidation products can be used for evaluating the stability of D vitamers under the condition of a “non-original matrix” or presence in complex matrices, in general.

In addition, an EC-MS approach offers the possibility of detecting reactive metabolites and allows targeted follow-up reactions (adduct formation or phase-II metabolism). For example, it could be possible to detect and identify reactive metabolites which would react further in vivo and/or be bound to other molecules that are present. Lohmann and Karst successfully simulated the metabolism of reactive metabolites like amodiaquine [29]. Furthermore, it was possible to verify the metabolism of tetrazepam based on electrochemical simulation [16]. All in all, the approach could be a promising tool to support animal studies for the investigation of phase-I and phase-II metabolism.

## Figures and Tables

**Table 1:** Summary of quasimolecular ions of detected oxidation products EC1–EC4 and CC1–CC4.

Substance	Quasimolecular Ion [M + H]^+^
ergocalciferol	397.1
EC1	431.1
EC2	411.1
EC3	427.1
EC4	427.1
cholecalciferol	385.1
CC1	419.1
CC2	399.1
CC3	415.5
CC4	415.1
